# Higher oxidative balance score is associated with a decreased risk of infertility: an analysis of NHANES 2013–2020

**DOI:** 10.3389/fnut.2025.1493253

**Published:** 2025-02-04

**Authors:** Mingjun Ma, Huanying Xu, Kexin Wang, Yanfen Chen, Ting Pan, Qiaoling Zhu

**Affiliations:** ^1^Foshan Clinical Medical School of Guangzhou University of Chinese Medicine, Foshan, Guangdong, China; ^2^Department of TCM Gynecology, Foshan Fosun Chancheng Hospital, Foshan, Guangdong, China

**Keywords:** oxidative balance score, female infertility, dietary antioxidants, oxidative stress, NHANES

## Abstract

**Background:**

Oxidative stress plays a crucial role in the female reproductive system. The oxidative balance score (OBS) is a new measure for assessing the balance between antioxidant and pro-oxidative factors in diet and lifestyle. However, limited studies have explored the relationship between OBS and infertility.

**Methods:**

We performed a cross-sectional study including 2,664 women aged 20–45 years, based on data from the 2013–2020 National Health and Nutrition Examination Survey (NHANES). The OBS was derived from 16 dietary components and 4 lifestyle factors. We used multivariate logistic regression analysis to examine the association between OBS and infertility.

**Results:**

The analysis revealed a significant negative association between higher OBS and infertility risk, with an odds ratio (OR) of 0.98 (95% CI, 0.95–1.00) after full adjustment. Compared to the first quartile of OBS, the second, third, and fourth quartiles showed ORs of 0.71 (95% CI, 0.45–1.11), 0.79 (95% CI, 0.51–1.22), and 0.57 (95% CI, 0.35–0.92), respectively. Similarly, women in the highest dietary OBS and lifestyle OBS quartiles had a lower infertility risk compared to those in the lowest quartiles, with ORs of 0.60 (95% CI, 0.39–0.94) and 0.54 (95% CI, 0.32–0.93), respectively. Furthermore, subgroup analysis indicated that the association between the fourth quartile of OBS and infertility remained consistent, except among women with other ethnicities—including multi-racial, and college graduate or higher.

**Conclusion:**

These findings suggest that high dietary OBS and lifestyle OBS are associated with a lower risk of infertility.

## Graphical Abstract

This study included a total of 2,664 women aged 20–45 years from National Health and Nutrition Examination Survey (NHANES) 2013–2020. Oxidative balance score (OBS), derived from 16 dietary and 4 lifestyle components, was analyzed to assess its association with self-reported infertility using multivariate logistic regression. The results indicate a significant inverse relationship between OBS and infertility risk. Higher dietary and lifestyle OBS also correlated with reduced infertility risk. NHANES, National Health and Nutrition Examination Survey; OBS, Oxidative balance score. 
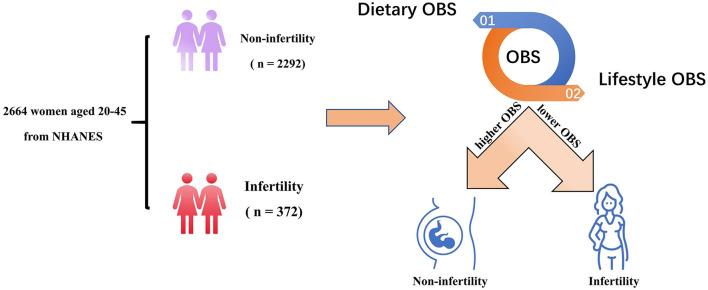

## Introduction

The World Health Organization (WHO) defines infertility as the failure to achieve pregnancy after 1 year or more of regular, unprotected sexual intercourse (1). Recent studies report that approximately 15% of couples of reproductive age have infertility, with rates reaching up to 30% in certain regions, including sub-Saharan Africa and South Asia, North Africa, the Middle East, Central and Eastern Europe, and Central Asia (2, 3). The causes of infertility are complex and involve various prevalent conditions. Several common diseases may affect female infertility, including premature ovarian insufficiency (4), polycystic ovary syndrome (5), endometriosis (6, 7), tubal obstruction (8), and chronic inflammatory diseases (4). Apart from these common diseases, lifestyle factors can also significantly influence female fertility, either positively or negatively (9). Adverse lifestyle choices, such as malnutrition, excessive alcohol consumption, smoking, excessive exercise, stress, inadequate sleep, environmental pollution, and unhealthy sexual practices, have been shown to negatively impact women's reproductive health and fertility (10). Due to the multiple factors influencing female fertility and the unclear underlying mechanisms, approximately 15% of infertile couples are diagnosed with “unexplained infertility” (11). Numerous studies have indicated that reactive oxygen species (ROS) production might contribute to infertility. ROS may play a role in multiple infertility-related pathological processes, such as peritoneal issues, tubal dysfunction, endometriosis, and unexplained infertility (12).

Oxidative stress describes a state of imbalance between the generation of ROS and the body's antioxidant defense mechanisms (13). It plays a crucial role in reproductive systems, affecting oocyte maturation in females, embryonic development, and implantation (14). Various factors influence the oxidative stress state, including the consumption of oxidative or antioxidant-rich foods (15–17) and lifestyle factors such as alcohol consumption, smoking, obesity, and physical activity (18, 19). Therefore, to comprehensively investigate the impact of dietary and lifestyle changes on oxidative stress status, it is particularly important to propose a method capable of integrating and quantifying the balance between antioxidant and pro-oxidant components.

The oxidative balance score (OBS) is a novel measure to assess the balance between antioxidant and pro-oxidative components in dietary and lifestyle factors (20). In general, a higher OBS indicates a preference for antioxidants over pro-oxidants (21). Multiple epidemiological investigations have shown a negative relationship between OBS and the likelihood of various common diseases, including hypertension, diabetes, and erectile dysfunction (22–24). However, the relationship between OBS and female infertility has been directly examined in only a limited number of studies.

Therefore, our study aims to explore the relationship between OBS and female infertility through a cross-sectional analysis of data from the National Health and Nutrition Examination Survey (NHANES) collected between 2013 and 2020. The OBS incorporated both lifestyle and dietary components. We hypothesized that higher OBS is associated with lower risk of female infertility, with the goal of providing new insights into the role of nutrition and lifestyle in female infertility and contributing to a broader understanding of how dietary and lifestyle factors influence reproductive health.

## Methods

### Participants and study design

Participants were selected from the NHANES, a stratified, multi-stage cluster probability sampling survey conducted during 2013–2020. The NHANES program provides a sample that represents the national population, and the data can be freely accessed on their official website (https://www.cdc.gov/nchs/nhanes/index.htm). NHANES studies are conducted every 2 years to assess the health and nutritional status of participants. All NHANES studies involving human participants are approved by the National Center for Health Statistics Ethics Review Board, and informed consent is obtained from all participants.

**Figure 1:**
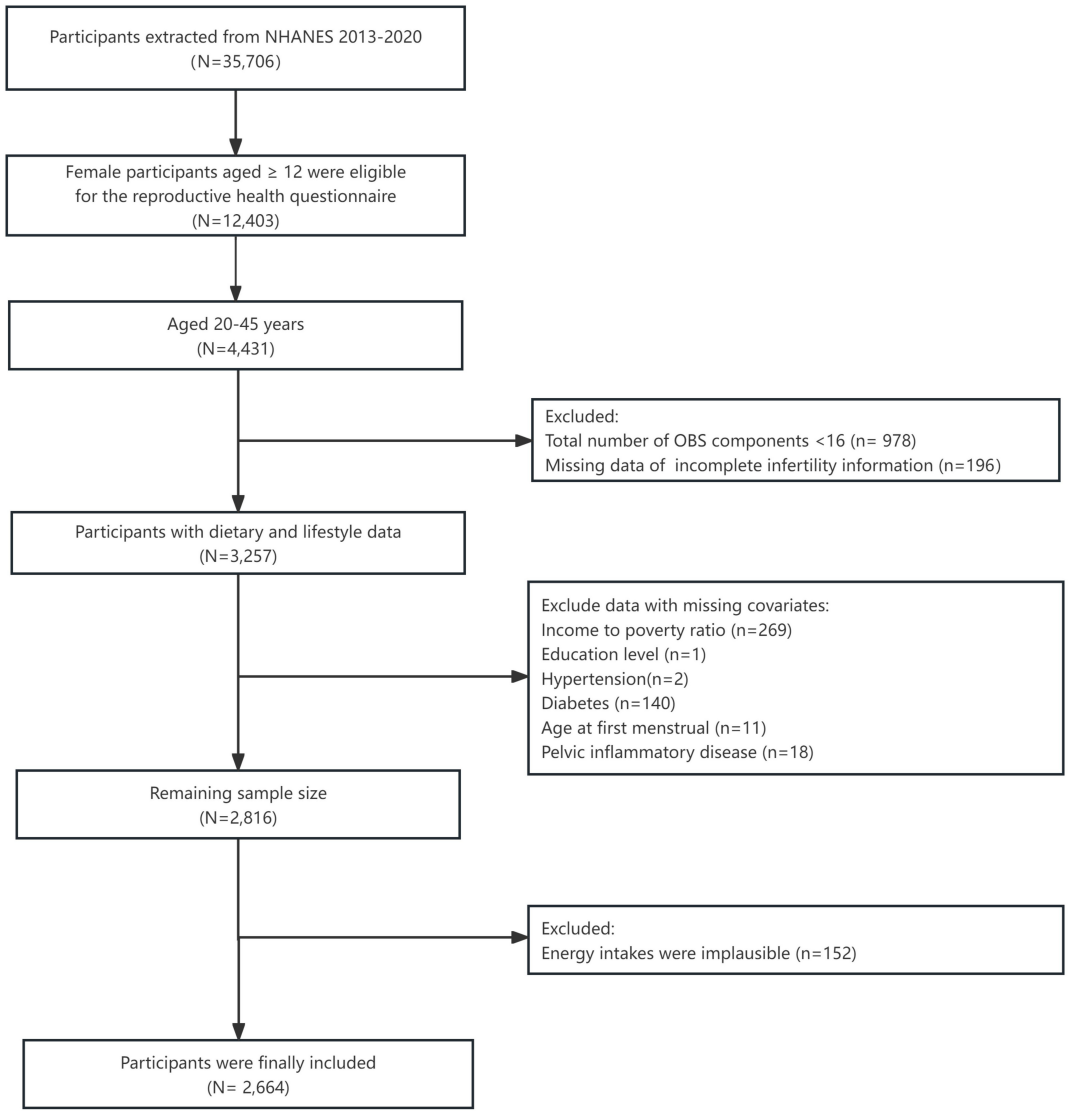
Flow chart of the study participants. NHANES, National Health and Nutrition Examination Survey.

Ultimately, 12,403 participants aged 12 years and above qualified to participate in the reproductive health questionnaire. However, our study exclusively included those between 20 and 45 years (*n* = 4,413) (25, 26). Participants were excluded from the final analysis based on the following criteria: (1) missing data in infertility information and OBS components (*N* = 1,174); (2) missing data in covariates (*N* = 441): including poverty income ratio (PIR), educational level, hypertension, diabetes, age at first menstrual period, and pelvic inflammatory disease (PID) and (3) participants who had tumors and implausible energy intakes (below 800 kcal/d or over 4,200 kcal/d) (*N* = 152). Ultimately, we selected 2,664 female participants in the final analysis (Figure 1).

### Oxidative balance score

The OBS was calculated by integrating 16 dietary components and 4 lifestyle factors, including 5 pro-oxidants and 15 antioxidants factors (27). Dietary OBS, including total folate, carotene, vitamins B6, B12, C, and E, niacin, riboflavin, dietary fiber, calcium, magnesium, zinc, copper, selenium, iron, and total fat, was obtained from two 24-hour dietary interviews. Antioxidants (such as vitamins and minerals) were scored based on tertiles 1 to 3, with scores of 0, 1, and 2, respectively. Conversely, pro-oxidants (iron and total fat) were scored in reverse order, with higher values receiving lower scores (28) (Supplementary Table S1). The lifestyle OBS incorporated four components: body mass index (BMI), cotinine, physical activity, and alcohol intake. Serum cotinine concentrations were measured to assess smoking exposure, covering both active smoking and second-hand smoke exposure (29). Physical activity in total was measured by applying the metabolic equivalent of task (MET) (30), derived from the total weekly duration of transportation, moderate, and intense activities. BMI, cotinine, and physical activity were scored based on tertiles 1 to 3, with scores of 0, 1, and 2 assigned, respectively. Alcohol intake is scored as 2, 1, and 0 points for non-drinkers (0 to 15 g/d), moderate drinkers, and heavy drinkers (≥15 g/d), respectively (28) (Supplementary Table S1). The total OBS is calculated by summing the scores of all components. An elevated OBS reflects a stronger antioxidant effect. Participants in this study were required to have a minimum of 16 out of the 20 OBS components (31).

### Self-reported infertility

Infertility was evaluated using two questions from the self-reported health survey (32): (1) RHQ074: “Have you ever attempted to conceive for a period of at least 1 year without success?” and (2) RHQ076: “Have you ever sought medical advice or consulted a healthcare professional because you were unable to conceive?” Women who answered “yes” to either of these questions were classified as having a history of infertility.

### Covariates

According to clinical practice and previous studies (32–34), our study used age (< 35, ≥35), race (including Mexican–American, other Hispanic, Non-Hispanic Black, Non-Hispanic White, and other Race—including Multi-Racial), marital status (Never married, Divorced/Separated/Widowed, and Married/Living with partner), education level (High school/GED/ < 11th grade, Some college or AA degree, and College graduate or above), income-to-poverty ratio (PIR), hypertension, diabetes, hyperlipidemia, pelvic inflammatory disease (PID), and age of menarche as covariates. The family PIR scale, ranging from 0 to 5, is divided into three categories. PID was determined through self-reported responses to questions from the reproductive health questionnaire, specifically: “Have you ever been treated for pelvic inflammatory disease/PID?” Age of menarche was divided into two categories: under 15 years and 15 years or older.

### Statistical analyses

All analyses were conducted based on the principle of complex multi-stage stratified sampling. Individual sample weight was computed by dividing the number of survey cycle years by the total number of survey cycle years and multiplying by the 2-day dietary sample weight (WTDR2D), following the recommended weight method in NHANES guidelines. Categorical variables were expressed as frequencies and percentages, while continuous variables were represented by means and standard deviations (SD). The Pearson chi-square test was used to analyze the differences in categorical variables between infertile and non-infertile female. Multivariate logistic regression was performed with different models to explore the association between OBS and infertility. Model 1 was a crude model. Model 2 was adjusted for age, race, education level, and marital status, and income-to-poverty ratio (PIR). To further exclude the effects of diabetes, hypertension, and hyperlipidemia, Model 3 was adjusted for Model 2 + diabetes, hypertension, and hyperlipidemia. Model 4 was adjusted for Model 3 + PID, and age of menarche. The OBS was also converted to a categorical variable, as well as being analyzed as a continuous variable.

Finally, sensitivity analyses were conducted to (1) explore the independent relationships between dietary OBS, lifestyle OBS, and infertility and (2) perform subgroup analyses stratified by factors such as race, pelvic inflammatory disease, and the diagnoses of hypertension, hyperlipidemia, and diabetes. The results of the interaction are presented as P-values. Data management and analyses were conducted using R version 4.1.2 (http://www.R-project.org). A two-tailed P-value of < 0.05 was regarded as statistically significant.

## Results

### Characteristics of the study population

A total of 2664 women aged 20–45 years were included in this study, of whom 13.96% (372/2,664) had infertility. Table 1 lists the baseline characteristics of the study participants. The average mean (SD) BMI of infertile and non-infertile women was 31.98 (0.73) and 28.92(0.32) kg/m^2^, respectively, and the mean (SD) age was 34.96 (0.56) and 32.24 (0.24) years, respectively. Women with infertility were generally older and showed a greater prevalence of obesity compared to non-infertile women (*P* < 0.05). Women without infertility had a lower proportion of being married or living with a partner compared to those with infertility (57.36% vs. 72.86%, *P* < 0.001). Infertile women exhibited a higher prevalence of PID history compared to non-infertile women (*P* < 0.05). There were no significant differences between the two groups in terms of race, PIR, education level, and age at first menstrual period.

**Table 1:** Characteristics of U.S. women aged 20–45 years from 2013 to 2020 NHANES, weighted.^*^

**Variable**	**Total**	**Non-infertility, *n* (%)**	**Infertility, *n* (%)**	***p*-value**
Overall, *N*	2,664	2,292 (86.04)	372 (13.96)	
**Age, years**, ***n*** **(%)**				< 0.001
< 35	1,436 (56.31)	1,285 (58.46)	151 (43.48)	
≥35	1,228 (43.69)	1,007 (41.54)	221 (56.52)	
**BMI, kg/m**^2^, ***n*** **(%)**				0.002
< 25.0	877 (36.63)	773 (38.23)	104 (28.05)	
25.0–30.0	616 (23.54)	554 (24.52)	62 (18.33)	
≥30.0	1,154 (39.43)	953 (37.25)	201 (53.62)	
**Race**, ***n*** **(%)**				0.810
Non-Hispanic White	914 (57.89)	772 (57.46)	142 (60.45)	
Non-Hispanic Black	636 (12.89)	549 (12.84)	87 (13.22)	
Mexican–American	416 (11.98)	364 (12.17)	52 (10.80)	
Other Hispanic	264 (7.02)	231 (7.05)	33 (6.86)	
Other Race—Including Multi-Racial	434 (10.22)	376 (10.48)	58 (8.68)	
**Marital status**, ***n*** **(%)**				< 0.001
Married/Living with partner	1,521 (59.58)	1,253 (57.36)	268 (72.86)	
Never married	853 (30.64)	790 (33.10)	63 (15.96)	
Divorced/Separated/Widowed	290 (9.78)	249 (9.54)	41 (11.17)	
**Education level**, ***n*** **(%)**				0.380
College graduate or above	786 (35.82)	674 (36.25)	112 (33.25)	
Some college or AA degree	1,009 (36.35)	865 (35.62)	144 (40.70)	
High school/GED/ < 11th grade	869 (27.83)	753 (28.13)	116 (26.04)	
**Income-to-poverty ratio**, ***n*** **(%)**				0.060
< 1.5	1,089 (31.72)	961 (32.66)	128 (26.08)	
1.5–3.5	842 (31.61)	717 (30.34)	125 (39.23)	
≥3.5	733 (36.67)	614 (37.00)	119 (34.69)	
**Hyperlipidemia**, ***n*** **(%)**				0.020
No	1,263 (49.01)	1,112 (50.76)	151 (38.59)	
Yes	1,401 (50.99)	1,180 (49.24)	221 (61.41)	
**Diabetes**, ***n*** **(%)**				0.001
No	2,504 (95.01)	2,169 (95.72)	335 (90.75)	
Yes	160 (4.99)	123 (4.28)	37 (9.25)	
**Hypertension**, ***n*** **(%)**				0.010
No	2,219 (86.95)	1,933 (88.04)	286 (80.42)	
Yes	445 (13.05)	359 (11.96)	86 (19.58)	
Pelvic infection, *n* (%)				0.002
No	2,530 (95.48)	2,190 (96.37)	340 (90.14)	
Yes	134 (4.52)	102 (3.63)	32 (9.86)	
**Age at first menstrual, years**, ***n*** **(%)**				0.630
< 15	2,198 (82.90)	1,894 (82.67)	304 (84.26)	
≥15	466 (17.10)	398 (17.33)	68 (15.74)	

^*^All estimates accounted for sample weights and complex survey designs, and percentages were adjusted for survey weights of NHANES.

BMI, body mass index; GED, general educational development; NHANES, National Health and Nutrition Examination Survey.

### Correlation between OBS and infertility

Table 2 demonstrates a negative association between OBS and infertility, and this relationship remains stable across various models. The OR of OBS for infertility was 0.98 (95% CI, 0.95–1.00) in the crude model. The results were robust in Model 2 (adjusting for age, race, marital status, education level, and PIR), Model 3 (adjusting for age, race, marital status, education level, PIR, hypertension, hyperlipidemia, and diabetes), and fully adjusted Model 4 (adjusting for age, race, marital status, PIR, hypertension, hyperlipidemia, diabetes, PID, and age of menarche), with ORs of 0.97 (95% CI, 0.95–1.00), 0.98 (95% CI, 0.95–1.00), and 0.98 (95% CI, 0.95–1.00), respectively. Then, we categorized OBS into quartiles to examine the possible relationship between OBS and infertility. Following full adjustment, the ORs for OBS levels in quartiles two, three, and four were 0.71 (95% CI, 0.45–1.11), 0.79 (95% CI, 0.51–1.22), and 0.57 (95% CI, 0.35–0.92), respectively, compared to the first quartile as the reference (p for trend = 0.04).

**Table 2:** Association between OBS and the risk of infertility in U.S. women aged 20–45 years from 2013 to 2020 NHANES, weighted.

	**Total, *n***	**Infertility, *n***	**Model 1**		**Model 2**		**Model 3**		**Model 4**	
			**OR (95% CI)**	***p*** **-value**	**OR (95% CI)**	***p*** **-value**	**OR(95% CI)**	***p*** **-value**	**OR(95% CI)**	***p*** **-value**
OBS (continuous)	2,664	372	0.98 (0.95, 1.00)	0.080	0.97 (0.95, 1.00)	0.040	0.98 (0.95, 1.00)	0.060	0.98 (0.95, 1.00)	0.080
**OBS (categories)**										
Quartile 1(3.00, 15.00)	695	111	ref		ref		ref		ref	
Quartile 2 (16.00, 21.00)	736	103	0.69 (0.45, 1.07)	0.100	0.66 (0.43, 1.02)	0.060	0.68 (0.43, 1.07)	0.100	0.71 (0.45, 1.11)	0.130
Quartile 3 (22.00, 26.00)	660	92	0.75 (0.49, 1.16)	0.190	0.74 (0.48, 1.13)	0.160	0.75 (0.48, 1.18)	0.210	0.79 (0.51, 1.22)	0.280
Quartile 4 (27.00, 37.00)	573	66	0.57 (0.35, 0.92)	0.020	0.53 (0.32, 0.86)	0.010	0.55 (0.33, 0.90)	0.020	0.57 (0.35, 0.92)	0.020
*P* for trend	NA	NA	0.040		0.020		0.030		0.040	

CI, confidence interval; OR, odds ratio; NHANES, National Health and Nutrition Examination Survey; OBS, oxidative balance score; Ref, reference group.

Model 1 was unadjusted.

Model 2 was adjusted for age (Continuous), race (Non-Hispanic White, Non-Hispanic Black, Mexican–American, Other Hispanic, Other Race—Including Multi-Racial), marital status (Married/Living with partner, Never married, Divorced/Separated/Widowed), education level (College graduate or above, Some college or AA degree, High school/GED/Less than 11th grade), and income-to-poverty ratio (< 1.5, 1.5-3.5, ≥3.5).

Model 3 was adjusted for Model 2 + Hypertension (No, Yes), Hyperlipidemia (No, Yes), and Diabetes (No, Yes).

Model 4 was adjusted for Model 3 + Pelvic infection (No, Yes), and Age of menarche (< 15, ≥15).

### Relationship between dietary/lifestyle OBS and infertility

An inverse association between dietary and lifestyle OBS and infertility is indicated in Table 3, and this relationship remains consistent across different models. In the crude model (Model 1), continuous lifestyle OBS demonstrated a significant negative association with female infertility, with an OR of 0.86 (95% CI: 0.78–0.94). In Models 3 and 4, the results were stable, with an OR of 0.88 (95% CI: 0.88–0.96) and 0.89 (95% CI: 0.81–0.97). Moreover, when dietary OBS and lifestyle OBS were categorized based on quartiles, the negative association with female infertility was found to be stronger for the highest quartile (Q4) of dietary OBS and lifestyle OBS compared to the lowest quartile (Q1). In dietary OBS Model 4, the OR for Q4 was 0.60 (95% CI: 0.39–0.94), while the ORs for infertility in Q2 and Q3 were 0.62 (95% CI: 0.40–1.95) and 0.97 (95% CI: 0.65–1.45), respectively (p for trend = 0.119). In lifestyle OBS Model 4, Q4 had an OR of 0.54 (95% CI: 0.32–0.93), while Q2 and Q3 had an OR of 1.05 (95% CI: 0.70–1.59) and 0.69 (95% CI: 0.44–1.09), respectively (*p* for trend = 0.009).

**Table 3:** Relationship between dietary/lifestyle OBS and infertility.

	**Total, *n***	**Infertility, *n***	**Model 1**		**Model 2**		**Model 3**		**Model 4**	
			**OR (95% CI)**	***p*** **-value**	**OR (95% CI)**	***p*** **-value**	**OR (95% CI)**	***p*** **-value**	**OR (95% CI)**	***p*** **-value**
**Dietary OBS (continuous)**	266,4	372	0.98 (0.96, 1.01)	0.240	0.98 (0.96, 1.01)	0.180	0.98 (0.95, 1.01)	0.170	0.98 (0.96, 1.01)	0.180
**Dietary OBS (categories)**										
Quartile 1 (2.00, 11.00)	681	105	ref		ref		ref		ref	
Quartile 2 (12.00, 17.00)	778	102	0.61 (0.40, 0.92)	0.020	0.59 (0.39, 0.90)	0.020	0.59 (0.38, 0.91)	0.020	0.62 (0.40, 0.95)	0.030
Quartile 3 (18.00, 21.00)	551	88	0.91 (0.59, 1.40)	0.660	0.94 (0.62, 1.43)	0.770	0.94 (0.62, 1.43)	0.770	0.97 (0.65, 1.45)	0.880
Quartile 4 (22.00, 31.00)	654	77	0.63 (0.40, 1.00)	0.050	0.59 (0.37, 0.95)	0.030	0.58 (0.37, 0.92)	0.020	0.60 (0.39, 0.94)	0.030
P for trend	NA	NA	0.164		0.125		0.106		0.119	
**Lifestyle OBS (continuous)**	266,4	372	0.86 (0.78, 0.94)	0.002	0.87 (0.80, 0.96)	0.004	0.88 (0.80, 0.96)	0.01	0.89 (0.81, 0.97)	0.010
**Lifestyle OBS (categories)**										
Quartile 1(0.00, 3.00)	997	167	ref	ref	ref	ref
Quartile 2 (4.00, 4.00)	495	67	1.00 (0.66, 1.52)	0.990	1.04 (0.69, 1.58)	0.840	0.99 (0.66, 1.48)	0.950	1.05 (0.70, 1.59)	0.800
Quartile 3 (5.00, 5.00)	630	80	0.65 (0.42, 1.01)	0.050	0.68 (0.44, 1.06)	0.090	0.66 (0.43, 1.03)	0.070	0.69 (0.44, 1.09)	0.110
Quartile 4 (6.00, 7.00)	542	58	0.46 (0.26, 0.81)	0.010	0.51 (0.29, 0.89)	0.020	0.52 (0.30, 0.89)	0.020	0.54 (0.32, 0.93)	0.030
*P* for trend	NA	NA	0.002		0.006		0.005		0.009	

OR, odds ratio; CI, confidence interval; OBS, oxidative balance score; Ref, reference group.

Model 1 was unadjusted.

Model 2 was adjusted for age (Continuous), race (Non-Hispanic White, Non-Hispanic Black, Mexican–American, Other Hispanic, Other Race—Including Multi-Racial), marital status (Married/Living with partner, Never married, Divorced/Separated/Widowed), education level (College graduate or above, Some college or AA degree, High school/GED/Less than 11th grade), and income-to-poverty ratio (< 1.5, 1.5-3.5, ≥3.5).

Model 3 was adjusted for Model 2 + Hypertension (No, Yes), Hyperlipidemia (No, Yes), and Diabetes (No, Yes).

Model 4 was adjusted for Model 3 + Pelvic infection, and Age of menarche (< 15, ≥15).

### Subgroup analysis

To confirm the stability of the association between OBS and infertility across different subgroups, we conducted subgroup analyses and interaction tests (Table 4). The findings indicated that the association between the fourth quartile of OBS and infertility remained generally stable across different subgroups, including age, race, marital status, education level, PIR, hyperlipidemia, diabetes, hypertension, PID, and age of menarche. In addition, the association between OBS and infertility was not influenced by the interactions across various subgroups (*P*-values for interaction > 0.05).

**Table 4:** Subgroup analyses of the association between OBS and infertility from 2013 to 2020 NHANES according to quartile of OBS.

	**Quartile 1 (3.00, 15.00)**	**Quartile 2 (16.00, 21.00)**	**Quartile 3 (22.00, 26.00)**	**Quartile 4 (27.00, 37.00)**	***P* for interaction**
**Age, years**, ***n*** **(%)**					0.960
< 35	Ref	0.77 (0.41, 1.43)	0.94 (0.46, 1.93)	0.69 (0.31, 1.50)	
≥35	Ref	0.65 (0.33, 1.25)	0.70 (0.37, 1.32)	0.48 (0.26, 0.91)	
**Race**, ***n*** **(%)**					0.560
Non-Hispanic White	Ref	0.59 (0.27, 1.29)	0.59 (0.31, 1.14)	0.44 (0.22, 0.88)	
Non-Hispanic Black	Ref	1.31 (0.68, 2.51)	1.47 (0.55,3.91)	0.83 (0.31, 2.24)	
Mexican–American	Ref	0.45(0.11, 1.92)	1.01 (0.29, 3.56)	0.31 (0.10, 0.98)	
Other Hispanic	Ref	0.48 (0.11, 2.13)	0.46 (0.11, 1.96)	0.52 (0.13, 2.02)	
Other Race—Including Multi-Racial	Ref	1.63 (0.42, 6.40)	3.24 (0.87, 12.10)	2.49 (0.73, 8.57)	
**Marital status**, ***n*** **(%)**					0.490
Living with partner	Ref	1.34 (0.62, 2.92)	1.58 (0.60, 4.17)	0.58 (0.11, 3.02)	
Never married	Ref	0.56 (0.31, 1.03)	0.69 (0.41, 1.17)	0.53 (0.31, 0.91)	
Divorced/Separated/Widowed	Ref	0.85 (0.25, 2.86)	0.60 (0.14, 2.61)	0.54 (0.11, 2.70)	
**Education level**, ***n*** **(%)**					0.780
College graduate or above	Ref	1.32 (0.38, 4.61)	1.56 (0.46, 5.29)	1.14 (0.37, 3.51)	
Some college or AA degree	Ref	0.80 (0.43, 1.49)	0.82 (0.43, 1.55)	0.50 (0.24, 1.06)	
High school/GED/ < 11th grade	Ref	0.50 (0.25, 0.98)	0.66 (0.31, 1.43)	0.41 (0.17,0.96)	
**Income-to-poverty ratio**, ***n*** **(%)**					0.680
< 1.5	Ref	0.72 (0.42, 1.23)	0.90 (0.41, 1.98)	0.68 (0.29, 1.58)	
1.5–3.5	Ref	0.54 (0.27, 1.10)	0.60 (0.32, 1.10)	0.69 (0.31, 1.53)	
≥3.5	Ref	0.90 (0.33, 2.48)	0.88 (0.34, 2.26)	0.45 (0.17, 1.16)	
**Hyperlipidemia**, ***n*** **(%)**					0.550
No	Ref	0.66 (0.34, 1.28)	0.54 (0.28, 1.06)	0.43 (0.20, 0.92)	
Yes	Ref	0.70 (0.38, 1.29)	1.00 (0.54, 1.83)	0.68 (0.38, 1.21)	
**Diabetes**, ***n*** **(%)**					0.160
No	Ref	0.74 (0.47, 1.16)	0.73 (0.45, 1.17)	0.55 (0.34, 0.90)	
Yes	Ref	0.70 (0.38, 1.29)	1.00 (0.54, 1.83)	0.68 (0.38, 1.21)	
**Hypertension**, ***n*** **(%)**					0.720
No	Ref	0.66 (0.40, 1.08)	0.82 (0.52, 1.30)	0.53 (0.31, 0.90)	
Yes	Ref	0.94 (0.37, 2.43)	0.55 (0.17, 1.76)	0.67 (0.24, 1.85)	
**Pelvic infection**, ***n*** **(%)**					0.950
No	Ref	0.71 (0.45, 1.13)	0.80 (0.51, 1.25)	0.56 (0.35, 0.92)	
Yes	Ref	1.00 (0.29, 3.48)	1.19 (0.13, 10.78)	1.00 (0.13, 7.71)	
**Age at first menstrual period, years**, ***n*** **(%)**					0.720
< 15	Ref	0.76 (0.48, 1.21)	0.78 (0.49, 1.23)	0.60 (0.35, 1.03)	
≥15	Ref	0.58 (0.20, 1.65)	1.10 (0.27, 4.45)	0.55 (0.15, 1.97)	

BMI, body mass index; CI, confidence interval; GED, general educational development; OBS, oxidative balance score; OR, odds ratios; Ref, reference group.

The model was adjusted for age (when not testing age), body mass index (when not testing body mass index), race (when not testing race), marital status (when not testing marital status), education level (when not testing education level), income-to-poverty ratio (when not testing income-to-poverty ratio), hyperlipidemia (when not testing hyperlipidemia), diabetes (when not testing diabetes), hypertension (when not Hypertension), pelvic infection (when not testing pelvic infection), and age at first menstrual period (when not testing age at first menstrual period).

## Discussion

In this nationally representative cross-sectional survey based on NHANES, a notable negative correlation was observed between OBS and female infertility. The higher total OBS was associated with a lower incidence of female infertility, with substantial trends across OBS quartiles. This negative association persisted even after adjusting for various covariates, indicating a 43% decrease in the odds of infertility from the first to the fourth quartile of OBS. Furthermore, in comparison with the lowest quartile, the highest quartiles of dietary OBS and lifestyle OBS were significantly associated with a lower risk of infertility. For dietary OBS, every single-unit increase was associated with an 11% reduction in infertility rates after full adjustment. Notably, lifestyle OBS demonstrated a more pronounced effect; every single-unit increase in lifestyle OBS corresponded to a 46% reduction in infertility rates after full adjustment.

OBS consists of 20 components, namely, total folate, carotene, vitamins (B6, B12, C, and E), niacin, riboflavin, dietary fiber, calcium, magnesium, iron, zinc, copper, selenium, total fat, BMI, smoking, alcohol intake, and physical activity, many of which have been shown to be closely associated with female infertility in previous studies. A cohort study of 2,370 women in the US population revealed a significant negative correlation between dietary fiber intake and female infertility (OR: 0.643, 95% CI: 0.480–0.861) (35). A randomized controlled trial called The Fast Track and Standard Treatment (FASTT) showed that higher intakes of antioxidants, including β-carotene, vitamin C, and vitamin E, were associated with a shorter time to conception among a cohort of couples being treated for unexplained infertility (36). Similarly based on a cohort of 18,555 married women, the Nurses' Health Study (37) included total intakes of each nutrient such as folic acid, various vitamins, iron, zinc, niacin, pantothenate, and retinol in the multivariable adjustment model to examine whether the inclusion of individual nutrients would attenuate the relationship between multivitamin use and infertility. The results showed that, after multivariable adjustment, the RR and 95% CI for infertility among women consuming more than six nutrients per week were 0.59 (95% CI: 0.46–0.75; *P* < 0.001), and the relationship between multivitamin use and infertility remained robust even after adjusting for individual nutrients such as iron, vitamin D, and folic acid. Furthermore, Dimitrios (38) indicated that calcium and vitamin D intake suppresses parathyroid hormone production, potentially improving hyperandrogenemia and anovulation associated with polycystic ovary syndrome (PCOS), thereby enhancing the chances of conception. In addition, a study conducted in a gynecology hospital in Nigeria involving 90 participants also found significantly lower levels of zinc (Zn) and magnesium (Mg) (*P* < 0.05) and significantly higher selenium (Se) levels (*P* < 0.05) in women with infertility compared to controls (39). Based on our findings, the quartile analysis of dietary OBS shows that higher quartiles of dietary OBS are significantly associated with a reduced risk of infertility compared to the lowest quartile (e.g., the OR for the fourth quartile is 0.60, 95% CI: 0.39–0.94, *P* = 0.030), further supporting the potential link between diet and reproductive health. These results suggest that improving diet quality, such as increasing the intake of antioxidants, dietary fiber, and key micronutrients, could guide public health interventions to reduce the incidence of infertility.

In addition to dietary factors, lifestyle is also associated with infertility. A descriptive cross-sectional study (40) involving 216 couples found significant differences in physical activity levels between infertile and fertile women (73.1% vs. 86.1%, respectively; *p* = 0.03). A systematic review of 98,657 women reported a combined relative risk (RR) of 0.87 (95% CI: 0.78–0.95) for fecundability among alcohol consumers compared to non-drinkers (41). Similarly, Goldman (42) found an increase in infertility, due to ovulatory factor or endometriosis, with alcohol use. Specifically, compared to non-drinkers, the OR for ovulatory infertility was 1.3 (95% CI: 1.0–1.7) in moderate drinkers and 1.6 (95% CI: 1.1–2.3) in heavy drinkers. BMI has also been shown to influence infertility. Grodstein (43) reported that, compared to women with lower BMI (20–24.9), obese women (BMI ≥27) had a relative risk (RR) of 3.1 (95% CI: 2.2–4.4) for ovulatory infertility. Moreover, the risk of ovulatory infertility was slightly elevated in moderately overweight women (BMI 25–26.9) and underweight women (BMI < 17), with RRs of 1.2 (95% CI: 0.8–1.9) and 1.6 (95% CI: 0.7–3.9), respectively. In addition, a controlled clinical study identified a significant correlation between smoking and female fertility, demonstrating markedly higher cotinine levels in follicular fluid among active smokers (710.4 ± 128.2 ng/mL) compared to passive smokers (76.3 ± 56.5 ng/mL) and non-smokers (4.2 ± 2.0 ng/mL), highlighting the detrimental impact of smoking on female fertility (44). Based on our findings, the quartile analysis of lifestyle OBS shows that higher quartiles of lifestyle OBS are significantly associated with a reduced risk of infertility compared to the lowest quartile (e.g., the OR for the fourth quartile is 0.54, 95% CI: 0.32–0.93, *P* = 0.030). This result further supports the importance of lifestyle factors, such as physical activity, BMI control, limiting alcohol consumption, and smoking cessation, in reproductive health. These findings suggest that improving lifestyle factors could provide effective public health interventions to reduce the incidence of infertility, particularly among high-risk populations.

Our findings suggest that infertility risk is associated with the combined oxidative and antioxidant profiles of multiple components. Compared to individual factors, OBS, as a composite index that includes both pro-oxidants and antioxidants, gives a more comprehensive reflection of the combined impact of diet and lifestyle on oxidative stress (45, 46). Therefore, the OBS is used as a comprehensive measure of exposure related to oxidative stress. Previous studies have shown that high OBS is associated with many other reproductive-related diseases. For example, a cross-sectional survey of the U.S. population indicated that elevated OBS was linked to a reduced prevalence of endometriosis, especially in women with irregular menstrual cycles and those taking female hormone supplements (47). Therefore, it is important to assess the relationship between oxidative balance and female infertility with composite indicators. These findings highlight the potential for using OBS as a framework for developing public health interventions or policies aimed at reducing infertility risk by promoting dietary and lifestyle improvements to maintain optimal oxidative balance.

Our study has several advantages. To the best of our knowledge, compared to previous studies that only focused on the effect of single dietary nutrient intake on female infertility, this study uses a more comprehensive dietary antioxidant capacity measurement method to evaluate the contribution of multiple dietary antioxidants to female infertility (31, 48). Moreover, compared to other dietary antioxidant composite indicators, this indicator includes a greater number of comprehensive factors and reasonably incorporates lifestyle factors (49, 50). Subgroup analysis and interaction tests were further performed on potential confounders such as age, race, marital status, education level, PIR, hypertension, diabetes, hyperlipidemia, PID, and age at menarche to confirm the robustness of the study results. This study included a nationally representative large sample that is reflective of the national population. The results can be extrapolated to the female infertility population nationwide, providing evidence-based dietary and lifestyle recommendations for female infertility management.

This study has several limitations. First, infertility has multifactorial causes, and we were unable to identify which specific type of infertility is most affected by the OBS. Future research should focus more on exploring the relationship between OBS and different types of infertility. Second, the cross-sectional design of the data limits the ability to establish a causal relationship between OBS and infertility, and only a correlation can be obtained. Third, the role of endogenous factors within the oxidative stress pathway requires further investigation. The absence of direct oxidative stress biomarkers in this study restricts the ability to validate the effectiveness of OBS and limits our understanding of the mechanisms underlying these observations. Fourth, the diagnosis of infertility was solely based on questionnaire data, without incorporating more specific clinical symptoms, which may lead to potential biases in the inclusion of study participants. Finally, our study primarily focused on European and American populations, which could limit the applicability of our results to other ethnic groups. Therefore, future research with larger and more ethnically diverse samples is essential to confirm our findings across various populations. Despite these limitations, our study preliminarily confirms the significant role of OBS in infertility, highlighting the importance of the synergistic effects of multiple factors. These findings offer valuable insights and serve as a foundation for guiding future research directions.

## Conclusion

Our findings suggest that high dietary OBS and lifestyle OBS are associated with a lower risk of infertility. These findings support the idea that improving diet and lifestyle factors in a comprehensive manner can reduce the risk of female infertility, particularly through increased dietary antioxidant intake and optimizing lifestyle factors such as smoking cessation, weight management, and regular physical activity to enhance reproductive health.

## Data Availability

The datasets presented in this study can be found in online repositories. The names of the repository/repositories and accession number(s) can be found below: https://www.cdc.gov/nchs/nhanes/index.htm.

## References

[1] World Health Organization. Infertility Prevalence Estimates: 1990–2021. Available at: https://www.who.int/publications/i/item/978920068315 (accessed April 3, 2023).

[2] Van Der Kelen A Okutman Ö Javey E Serdarogullari M Janssens C Ghosh MS. A systematic review and evidence assessment of monogenic gene-disease relationships in human female infertility and differences in sex development. Hum Reprod Update. (2023) 29:218–32. 10.1093/humupd/dmac044 36571510

[3] Mascarenhas MN Flaxman SR Boerma T Vanderpoel S Stevens GA. National, regional, and global trends in infertility prevalence since 1990: a systematic analysis of 277 health surveys. PLoS Med. (2012) 9:e1001356. 10.1371/journal.pmed.1001356 23271957 PMC3525527

[4] Hart RJ. Physiological aspects of female fertility: role of the environment, modern lifestyle, and genetics. Physiol Rev. (2016) 96:873–909. 10.1152/physrev.00023.2015 27252278

[5] Stener-Victorin E Teede H Norman RJ Legro R Goodarzi MO Dokras A. Polycystic ovary syndrome. Nature reviews Disease primers. (2024) 10:27. 10.1038/s41572-024-00511-3 38637590

[6] Tanbo T Fedorcsak P. Endometriosis-associated infertility: aspects of pathophysiological mechanisms and treatment options. Acta Obstet Gynecol Scand. (2017) 96:659–67. 10.1111/aogs.13082 27998009

[7] Barnhart K Dunsmoor-Su R Coutifaris C. Effect of endometriosis on in vitro fertilization. Fertil Steril. (2002) 77:1148–55. 10.1016/S0015-0282(02)03112-6 12057720

[8] El-Kharoubi AF Szasz F. Tubal blockage surgery: a retrospective cohort study on clinical characteristics and reproductive outcomes within six years. Cureus. (2023) 15:e39879. 10.7759/cureus.39879 37404391 PMC10315170

[9] Skoracka K Eder P Łykowska-Szuber L Dobrowolska A Krela-Kazmierczak I. Diet and nutritional factors in male (in)fertility-underestimated factors. J Clini Med. (2020) 9:1400. 10.3390/jcm9051400 32397485 PMC7291266

[10] Ferramosca A Zara V. Diet and male fertility: the impact of nutrients and antioxidants on sperm energetic metabolism. Int J Mol Sci. (2022) 23:2542. 10.3390/ijms23052542 35269682 PMC8910394

[11] Carson SA Kallen AN. Diagnosis and management of infertility: a review. JAMA. (2021) 326:65–76. 10.1001/jama.2021.4788 34228062 PMC9302705

[12] Anton C Ciobica A Doroftei B Maftei R Ilea C Darii Plopa N. A review of the complex relationship between irritable bowel syndrome and infertility. Medicina. (2020) 56:592. 10.3390/medicina56110592 33172048 PMC7694637

[13] Palomba S Daolio J Romeo S Battaglia FA Marci R La Sala GB. Lifestyle and fertility: the influence of stress and quality of life on female fertility. Reprod Biol Endocrinol. (2018) 16:113. 10.1186/s12958-018-0434-y 30501641 PMC6275085

[14] Qi L Li Y Zhang L Li S Zhang X Li W. Immune and oxidative stress disorder in ovulation-dysfunction women revealed by single-cell transcriptome. Front Immunol. (2023) 14:1297484. 10.3389/fimmu.2023.1297484 38116006 PMC10729704

[15] Agarwal A Gupta S Sharma RK. Role of oxidative stress in female reproduction. Reprod Biol Endocrinol. (2005) 3:28. 10.1186/1477-7827-3-28 16018814 PMC1215514

[16] Fang YZ Yang S Wu G. Free radicals, antioxidants, and nutrition. Nutrition. (2002) 18:872–9. 10.1016/S0899-9007(02)00916-4 12361782

[17] Valko M Rhodes CJ Moncol J Izakovic M Mazur M. Free radicals, metals and antioxidants in oxidative stress-induced cancer. Chem Biol Interact. (2006) 160:1–40. 10.1016/j.cbi.2005.12.009 16430879

[18] Albano E. Alcohol, oxidative stress and free radical damage. Proc Nutr Soc. (2006) 65:278–90. 10.1079/PNS2006496 16923312

[19] Barreiro E Peinado VI Galdiz JB Ferrer E Marin-Corral J Sánchez F. Cigarette smoke-induced oxidative stress: a role in chronic obstructive pulmonary disease skeletal muscle dysfunction. Am J Respir Crit Care Med. (2010) 182:477–88. 10.1164/rccm.200908-1220OC 20413628

[20] Jakubiak GK Osadnik K Lejawa M Kasperczyk S Osadnik T Pawlas N. Oxidative stress in association with metabolic health and obesity in young adults. Oxid Med Cell Longev. (2021) 2021:9987352. 10.1155/2021/9987352 34257828 PMC8257366

[21] Xu Z Xue Y Wen H Chen C. Association of oxidative balance score and lung health from the national health and nutrition examination survey 2007-2012. Front Nutrit. (2022) 9:961950. 10.3389/fnut.2022.961950 36698460 PMC9869685

[22] Hernández-Ruiz Á García-Villanova B Guerra-Hernández EJ Carrión-García CJ Amiano P Sánchez MJ. Oxidative balance scores (Obss) integrating nutrient, food and lifestyle dimensions: development of the nutrientl-Obs and foodl-Obs. Antioxidants. (2022) 11:300. 10.3390/antiox11020300 35204183 PMC8868253

[23] Lee JH Son DH Kwon YJ. Association between oxidative balance score and new-onset hypertension in adults: a community-based prospective cohort study. Front Nutr. (2022) 9:1066159. 10.3389/fnut.2022.1066159 36590204 PMC9798298

[24] Demirer B Yardimci H Erem Basmaz S. Inflammation level in type 2 diabetes is associated with dietary advanced glycation end products, mediterranean diet adherence and oxidative balance score: a pathway analysis. J Diabetes Complicat. (2023) 37:108354. 10.1016/j.jdiacomp.2022.108354 36493637

[25] Dong M Xu X Li Y Wang Y Jin Z Tan J. Impact of infertility duration on female sexual health. Reprod Biol Endocrinol. (2021) 19:157. 10.1186/s12958-021-00837-7 34627263 PMC8501599

[26] Tang J Xu Y Wang Z Ji X Qiu Q Mai Z. Association between metabolic healthy obesity and female infertility: the national health and nutrition examination survey, 2013-2020. BMC Public Health. (2023) 23:1524. 10.1186/s12889-023-16397-x 37563562 PMC10416469

[27] Wang L Huang S Feng Z Xiao J Luo G Zhang Y. Assessing the role of antioxidant and pro-oxidant balance in mediating the relationship between vitamin K intake and depressive symptoms in adults. Front Nutr. (2024) 11:1384489. 10.3389/fnut.2024.1384489 39027663 PMC11254852

[28] Zhang W Peng SF Chen L Chen HM Cheng XE Tang YH. Association between the oxidative balance score and telomere length from the national health and nutrition examination survey 1999-2002. Oxid Med Cell Longev. (2022) 2022:1345071. 10.1155/2022/1345071 35186180 PMC8850082

[29] Tsai J Homa DM Neff LJ Sosnoff CS Wang L Blount BC. Trends in secondhand smoke exposure, 2011-2018: impact and implications of expanding serum cotinine range. Am J Prev Med. (2021) 61:e109–e17. 10.1016/j.amepre.2021.04.004 34419235

[30] Wang Y Li J Sun W Tong Y Han L Jiang Z. Associations between the oxidative balance score and constipation: a cross-sectional study of the NHANES, 2005-2010. BMC Public Health. (2024) 24:1908. 10.1186/s12889-024-19428-3 39014407 PMC11253473

[31] Liu F You F Yang L Du X Li C Chen G. Nonlinear relationship between oxidative balance score and hyperuricemia: analyses of NHANES 2007-2018. Nutr J. (2024) 23:48. 10.1186/s12937-024-00953-1 38704549 PMC11069158

[32] Xu H Wen Q Xing X Chen Y Zhu Q Tan M. High dietary inflammatory index increases the risk of female infertility: an analysis of NHANES 2013-2018. Nutrition research (New York, NY). (2024) 125:50–60. 10.1016/j.nutres.2024.02.006 38503022

[33] Lyra P Botelho J Machado V Rota S Walker R Staunton J. Self-reported periodontitis and C-reactive protein in parkinson's disease: a cross-sectional study of two american cohorts. NPJ Parkinson's Dis. (2022) 8:40. 10.1038/s41531-022-00302-1 35418117 PMC9008053

[34] Chen Z Wu Z Zhang Y. Association between dietary magnesium intake and pelvic inflammatory disease in US women: a cross-sectional study of NHANES. Front Nutr. (2024) 11:1430730. 10.3389/fnut.2024.1430730 39171114 PMC11335488

[35] Cai Q Chen T. Association between dietary fiber and female fertility: a NHANES-based study. Reprod Sci. (2023) 30:1555–64. 10.1007/s43032-022-01103-w 36315393

[36] Ruder EH Hartman TJ Reindollar RH Goldman MB. Female dietary antioxidant intake and time to pregnancy among couples treated for unexplained infertility. Fertil Steril. (2014) 101:759–66. 10.1016/j.fertnstert.2013.11.008 24355050 PMC3943921

[37] Chavarro JE Rich-Edwards JW Rosner BA Willett WC. Use of multivitamins, intake of B vitamins, and risk of ovulatory infertility. Fertil Steril. (2008) 89:668–76. 10.1016/j.fertnstert.2007.03.089 17624345 PMC2366795

[38] Farmakiotis D Katsikis I Panidis D. Calcium homeostasis and anovulatory infertility. Hum Reprod. (2007) 22:3264. 10.1093/humrep/dem330 17933749

[39] Oluboyo A. Evaluation of selected trace elements and glutathione peroxidase levels in female infertility. J Obstet Gynecol Cancer Res. (2022) 7:374–81. 10.30699/jogcr.7.5.374

[40] Khosrorad T Dolatian M Riazi H Mahmoodi Z Alavimajd H Shahsavari S. Comparison of lifestyle in fertile and infertile couples in kermanshah during 2013. Iranian J Reprod Med. (2015) 13:549–56. 26568759 PMC4637122

[41] Fan D Liu L Xia Q Wang W Wu S Tian G. Female alcohol consumption and fecundability: a systematic review and dose-response meta-analysis. Sci Rep. (2017) 7:13815. 10.1038/s41598-017-14261-8 29062133 PMC5653745

[42] Grodstein F Goldman MB Cramer DW. Infertility in women and moderate alcohol use. Am J Public Health. (1994) 84:1429–32. 10.2105/AJPH.84.9.1429 8092366 PMC1615168

[43] Grodstein F Goldman MB Cramer DW. Body mass index and ovulatory infertility. Epidemiology. (1994) 5:247–50. 10.1097/00001648-199403000-00016 8173001

[44] Phipps WR Cramer DW Schiff I Belisle S Stillman R Albrecht B. The association between smoking and female infertility as influenced by cause of the infertility. Fertil Steril. (1987) 48:377–82. 10.1016/S0015-0282(16)59402-3 3114008

[45] Lakkur S Judd S Bostick RM McClellan W Flanders WD Stevens VL. Oxidative stress, inflammation, and markers of cardiovascular health. Atherosclerosis. (2015) 243:38–43. 10.1016/j.atherosclerosis.2015.08.032 26343870 PMC4609620

[46] Wen H Li X Chen J Li Y Yang N Tan N. Association of oxidative balance score with chronic kidney disease: NHANES 1999-2018. Front Endocrinol. (2024) 15:1396465. 10.3389/fendo.2024.1396465 38919480 PMC11198875

[47] Zhou X Shen W Zhu J Chen Y Zhang J. Association between the Oxidative Balance Score and Endometriosis: A Population-Based Study. Int J Women's Health. (2024) 16:1293–301. 10.2147/IJWH.S466189 39100109 PMC11297482

[48] Chen Q Bao W Kong X Zhu J Hou S Zhang Y. Association between the oxidative balance score and kidney stones in adults. World J Urol. (2024) 42:425. 10.1007/s00345-024-05144-5 39037613

[49] Jia Z Chen K. Association between the composite dietary antioxidant index and periodontitis in US adults: a cross-sectional analysis. Quint. Int. (2024) 55:734–42. 10.3290/j.qi.b5714863 39190015

[50] Lin W Lin J Lai F Shi J. Effect of dietary antioxidant quality score on tobacco smoke exposure and asthma in children and adolescents: a cross-sectional study from the NHANES database. BMC Pediatr. (2024) 24:535. 10.1186/s12887-024-05009-1 39169319 PMC11337629

