# Supplementary material for: Higher oxidative balance score is associated with a decreased risk of infertility: an analysis of NHANES 2013–2020
Source: Front Nutr. 2025 Feb 4;12:1493253. doi: 10.3389/fnut.2025.1493253 (PMC11832392; doi:10.3389/fnut.2025.1493253)
Supplement: Supplementary file 1 [file Table_1.docx]

Supplementary Material

# 1 Table S1 Oxidative balance score (OBS) **components** and score assignment.

| **OBS components** | **Property** | **0** | **1** | **2** |
| --- | --- | --- | --- | --- |
| **Dietary OBS components** | | | | |
| Dietary fiber (g/d) | A | <10.10 | 10.10-16.31 | ≥16.31 |
| Carotene (RE/d) | A | <98.08 | 98.08-383.50 | ≥383.50 |
| Riboflavin (mg/d) | A | <1.34 | 1.34-2.02 | ≥2.02 |
| Niacin (mg/d) | A | <14.52 | 14.52-21.86 | ≥21.86 |
| Vitamin B6 (mg/d) | A | <1.13 | 1.13-1.77 | ≥1.77 |
| Total folate (mcg/d) | A | <251.00 | 251.00-388.96 | ≥388.96 |
| Vitamin B12 (mcg/d) | A | <2.22 | 2.22-4.22 | ≥4.22 |
| Vitamin C (mg/d) | A | <38.01 | 38.01-98.49 | ≥98.49 |
| Vitamin E (ATE) (mg/d) | A | <4.53 | 4.53-7.52 | ≥7.52 |
| Calcium (mg/d) | A | <499.24 | 499.24-849.00 | ≥849.00 |
| Magnesium (mg/d) | A | <187.00 | 187.00-283.43 | ≥283.43 |
| Zinc (mg/d) | A | <6.73 | 6.73-10.75 | ≥10.75 |
| Copper (mg/d) | A | <0.85 | 0.85-1.28 | ≥1.28 |
| Selenium (mcg/d) | A | <67.79 | 67.79-99.50 | ≥99.50 |
| Total fat (g/d) | P | ≥50.98 | 50.98-75.79 | <75.79 |
| Iron (mg/d) | P | ≥9.65 | 9.65-14.32 | <14.32 |
| **Lifestyle OBS components** | | | | |
| Physical activity (MET-minute/week) | A | <270.00 | 270.00-845.71 | ≥845.71 |
| Alcohol (g/d) | P | ≥15 | 0-15 | None |
| Body mass index (kg/m2) | P | ≥23.74 | 23.74-28.64 | <28.64 |
| Cotinine (ng/mL) | P | ≥0.035 | 0.035-0.172 | <0.172 |

OBS: oxidative balance score; A: antioxidant; P: prooxidant; RE: retinol equivalent; ATE: alpha-tocopherol equivalent; MET: metabolic equivalent.
